# Relationship between maternal vitamin D status in the first trimester of pregnancy and maternal and neonatal outcomes: a retrospective single center study

**DOI:** 10.1186/s12887-021-02730-z

**Published:** 2021-07-29

**Authors:** Meng Ni, Qianqian Zhang, Jiuru Zhao, Qianwen Shen, Dongting Yao, Tao Wang, Zhiwei Liu

**Affiliations:** 1 International Peace Maternity and Child Health Hospital, School of Medicine, Shanghai Jiao Tong University, 910# Hengshan Road, Shanghai, 20030 China; 2 International Peace Maternity and Child Health Hospital of China Welfare Institution, School of Medicine, Shanghai Jiao Tong University, Shanghai, China; 3 Shanghai Key Laboratory of Embryo Original Disease, Shanghai, China

**Keywords:** Vitamin D, Maternal, Neonatal, Outcomes, NICU

## Abstract

**Background:**

This study aimed to investigate the relationship between maternal serum vitamin D status in the first trimester of pregnancy and maternal as well as neonatal outcomes, considered the prevalence of vitamin D deficiency (serum 25(OH)D < 50 nmol/L) around the world, especially in the pregnant women.

**Methods:**

From January 2015 to December 2016, in this cross-sectional retrospective study, we enrolled women receiving regular prenatal examinations and giving birth in the International Peace Maternity and Child Health Hospital. Cases confirmed as multiple pregnancy, incomplete medical records, and vitamin D level recorded after 13 weeks of gestation were excluded. A total of 23,394 mother-infant pairs were included ultimately. Obstetric and neonatal information were extracted from the database. Maternal serum vitamin D concentration was measured by chemiluminescence microparticle immunoassay. Logistic regression analysis (unadjusted and adjusted models) was used to analyze the association between vitamin D and maternal and neonatal outcomes.

**Results:**

The average 25(OH) D concentration was 43.20 ± 0.10 nmol/L; 67.09% of patients were vitamin D deficient(25(OH) D < 50.00 nmol/L), 29.84% were vitamin D insufficient (50 nmol/L ≤ 25(OH)D < 75 nmol/L), 3.07% were sufficient (25(OH)D ≥ 75 nmol/L). The maternal 25(OH)D levels varied with age, pre-pregnancy BMI, season when blood sample was collected, number of previous-pregnancy. Notably, newborns delivered by women with deficient vitamin D status had a higher incidence rate of admission to NICU (Deficiency: 12.20% vs Insufficiency: 10.90% vs Sufficiency: 11.70%, *P*_bonferroni_ = .002) and a longer stay (deficiency: 6.2 ± 4.1 days vs insufficiency: 5.9 ± 3.1 days vs sufficiency: 5.1 ± 2.1 days, *P*_bonferroni_ = .010). Moreover, maternal vitamin D deficiency was a dependent risk factor for admission to NICU (unadjusted OR = 1.35, 95% CI,1.05–1.74 *P*_bonferroni_ = .022; adjusted OR = 1.31, 95% CI,1.010–1.687 *P*_bonferroni_ = .042).

**Conclusions:**

Maternal vitamin D deficiency (25(OH) D < 50 nmol/L) was prevalent in eastern coastal China. The incidence rate of GDM as well as preeclampsia was higher in vitamin D insufficient group while vitamin D deficiency group was liable to intrauterine infection when compared with the other two groups. Most importantly, low vitamin D status in the first trimester of pregnancy was a dependent risk factor for admission to NICU. More well-designed perspective researches are necessary to clarify the role of vitamin D in the early stage of pregnancy.

## Introduction

Vitamin D, with a vital role in calcium absorption and bone metabolism, also functioned in cell proliferation and differentiation, affecting the immune system as well [1]. However, it’s worrisome that the incidence of vitamin D deficiency (VDD) is prevalent globally, especially in pregnant women [2]. Low vitamin D status (defined as serum 25(OH)D concentrations < 50 nmol/L) was found in 33% of pregnant women in the US and 24% in Canada, respectively. In Europe, the prevalence of low vitamin D status was from 20 to 77% [3]. According to the view of Developmental Origin of Health and Disease (DOHaD) theory, the disturbance in the uterine where the fetus is particularly sensitive to chemicals and other stressors, is related to adverse health effects in adult. Therefore, the lack of vitamin D may have effects on maternal and neonatal outcomes. Observational studies indicated an association between low vitamin D levels and an increased risk of disorders of placental implantation, impaired glucose tolerance, pre-eclampsia, fetal growth retardation, preterm birth, and caesarean section [4, 5], while Randomized Controlled Trial (RCT) found contradicted results [6–8]. Despite some short-term consequences in the newborns, maternal vitamin D deficiency also have long-term effects as rickets, increased susceptibility to respiratory illness, autoimmune diseases and type 1 diabetes [9, 10]. Considered the important but controversial role of vitamin D, we aimed to investigate the relationship between maternal vitamin D status in the first trimester of pregnancy and the outcomes of mothers and newborns in order to provide a practical recommendation for clinicians.

## Method

### Study design and participants

**Fig. 1:**
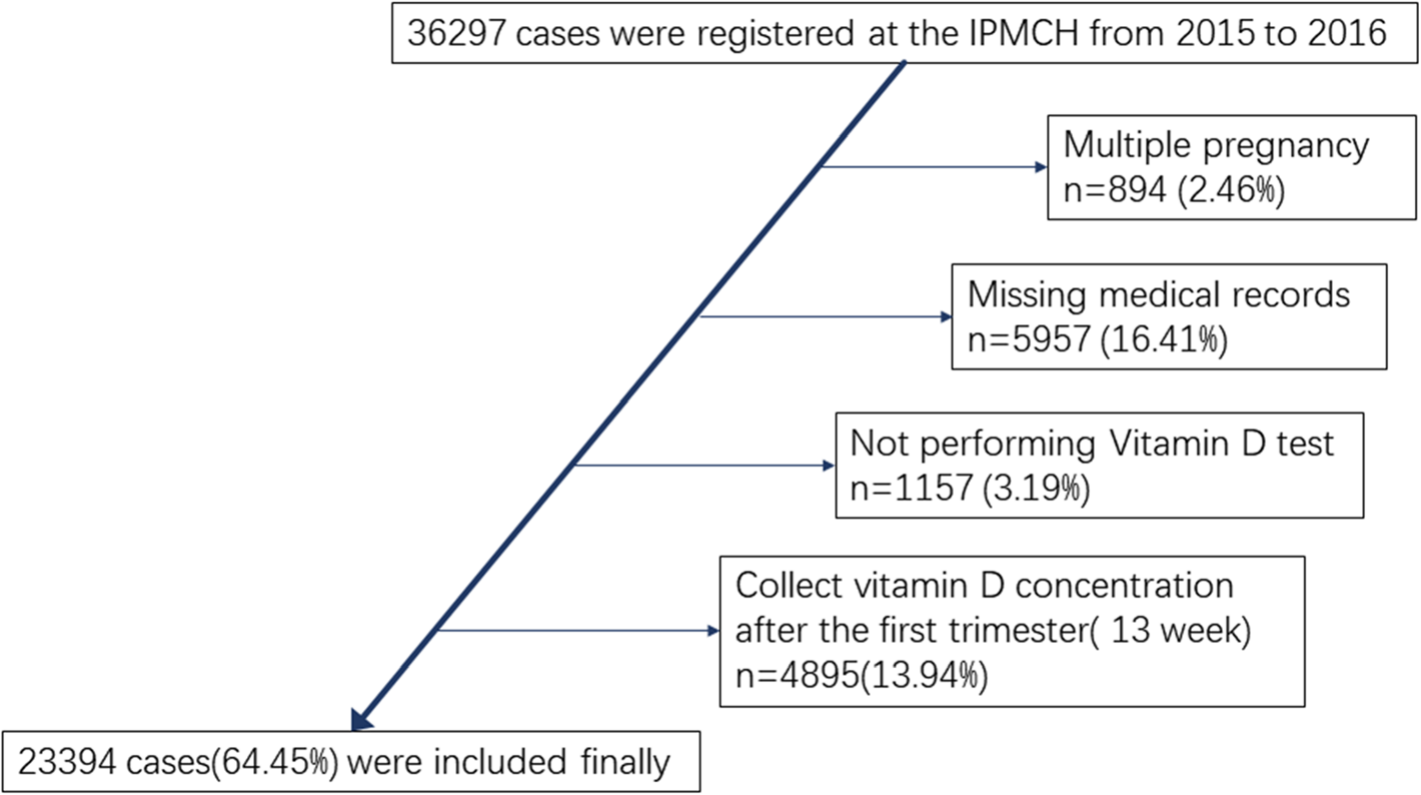
Flow chart of inclusion criterion of population

In this study, from 2015 to 2016, women followed regular antenatal examinations and gave birth at the International Peace Maternity and Child Health Hospital were included. After excluding cases with incomplete vitamin D and clinical information, diagnosis of multiple pregnancy, 23,394 mothers with their newborns were included ultimately (Fig. 1). Clinical data were extracted from the database and censored by two professional assistants respectively. When disagreement emerged, suggestions from an experienced clinician were considered. All diagnoses were confirmed according to clinical guidelines.

### Data collection

Demographic records were collected at the enrollment along with questionnaires. It consisted of maternal age, body mass index (BMI) before pregnancy, gravidity or parity. The calculation of gestational age was based on the date of the last menstrual period (MLP), then verified or adjusted by ultrasound reports. Seasons when samples were collected were classified as winter, spring, summer and fall.

### Vitamin D level assessment and classification

We collected maternal fasting blood samples at the first antenatal visit (9–13 weeks of gestation), then transferred it to hospital laboratory, certified by China National Accreditation Service for Conformity Assessment. The blood sample was centrifuged to obtain the serum and stored at 4 °C. Quantitative analysis of vitamin D was performed using chemiluminescence microparticle immunoassay in an Architect I2000SR automatic analyzer (Abbott Diagnostics) with a standard curve following standard clinical procedures by two qualified inspectors. The detection range of vitamin D was 2.00 to 400.00 nmol/L with both intra- and inter-assay coefficients of variation less than 5%. According to the Endocrine Society Clinical Practice Guideline [11], mothers with serum 25(OH)D concentration less than 50.00 nmol/L were classified as vitamin D deficiency, 50.00 to 75.00 nmol/L as insufficiency, and more than 75.00 nmol/L as sufficiency respectively.

### Maternal and neonatal outcomes

1. Maternal outcomes:preterm delivery (a live birth before 37 weeks of gestation), pre-eclampsia (high blood pressure and excess protein in the urine after 20 weeks of pregnancy), gestational diabetes (any of the following criteria are meet at a 75 g oral glucose tolerance test during 24–28 weeks of gestation: fasting: ⩾ 92 mg/dl,1 h: ⩾ 180 mg/dl, 2 h: ⩾153 mg/dl at a), Intrahepatic cholestasis (pruritus, elevated serum total bile acid (> 10 μmol/L) and/or alanine aminotransferase), intrauterine inflammation (pathologic diagnosis of placenta or clinical diagnosis (maternal fever, leukocytosis, maternal and/or fetal tachycardia, uterine tenderness, and preterm rupture of membranes (PROM)). 
2. Neonatal outcomes: birth weight and height, low birth weight (< 2500 g), macrosomia (> 4000 g), small-for-gestational-age (weight < 10th percentile or 2SD at birth), score of Apgar 5′, NICU hospitalization (for any reasons), asphyxia of newborn, asphyxia of newborn, hyperbilirubinemia, necrotizing enterocolitis, sepsis, death (for any reason).
3. Criterion for admission to NICU: All the preterm infants, the infants with severe complications, such as visual or hearing impairment, chronic lung disease, temperature instability, hypoglycemia, respiratory distress, hyperbilirubinemia or jaundice, feeding difficulties, urinary tract infection, diarrhea, meningitis and neonatal mortality. All the conditions were diagnosed according to clinical protocols and decisions were made by two experienced neonatologists when infants needed to be transferred to NICU.

### Statistical analysis

All the statistical analyses were performed by the software package SPSS (V25, IBM Corp, Armonk, NY, USA). Continuous data were presented as mean ± standard deviation (SD) while categorical ones as number (%). The normality of maternal vitamin D levels was determined by Kolmogorov-Smirnov test. Distribution of maternal demographic characteristics were compared among groups using the Kruskal—Wallis *H*-test, Pearson’s chi-square test or Fisher’s exact test based on data characteristic. Bonferroni corrections were applied for multiple comparison correction. Multivariate logistic regression analysis was applied to investigate the relationship between maternal vitamin D level and maternal and neonatal outcomes. The model was adjusted by maternal age, BMI before pregnancy, gestational weeks and season of blood collection (spring, summer, autumn, winter), delivery mode, number of pre-pregnancies. All *P* values were 2-tailed and *P* < 0.05 was defined as statistically significant. Odds ratios (ORs) and 95% confidence intervals (CIs) were applied for the unadjusted and adjusted models.

## Results

### General description of vitamin D status

**Fig. 2:**
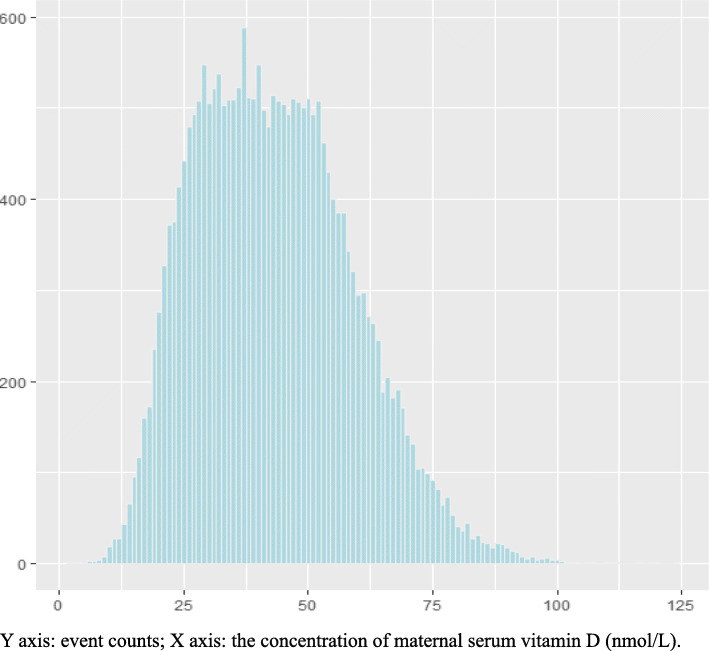
Distribution of maternal Vitamin D status in the first trimester of pregnancy. Y axis: event counts; X axis: the concentration of maternal serum vitamin D (nmol/L)

**Fig. 3:**
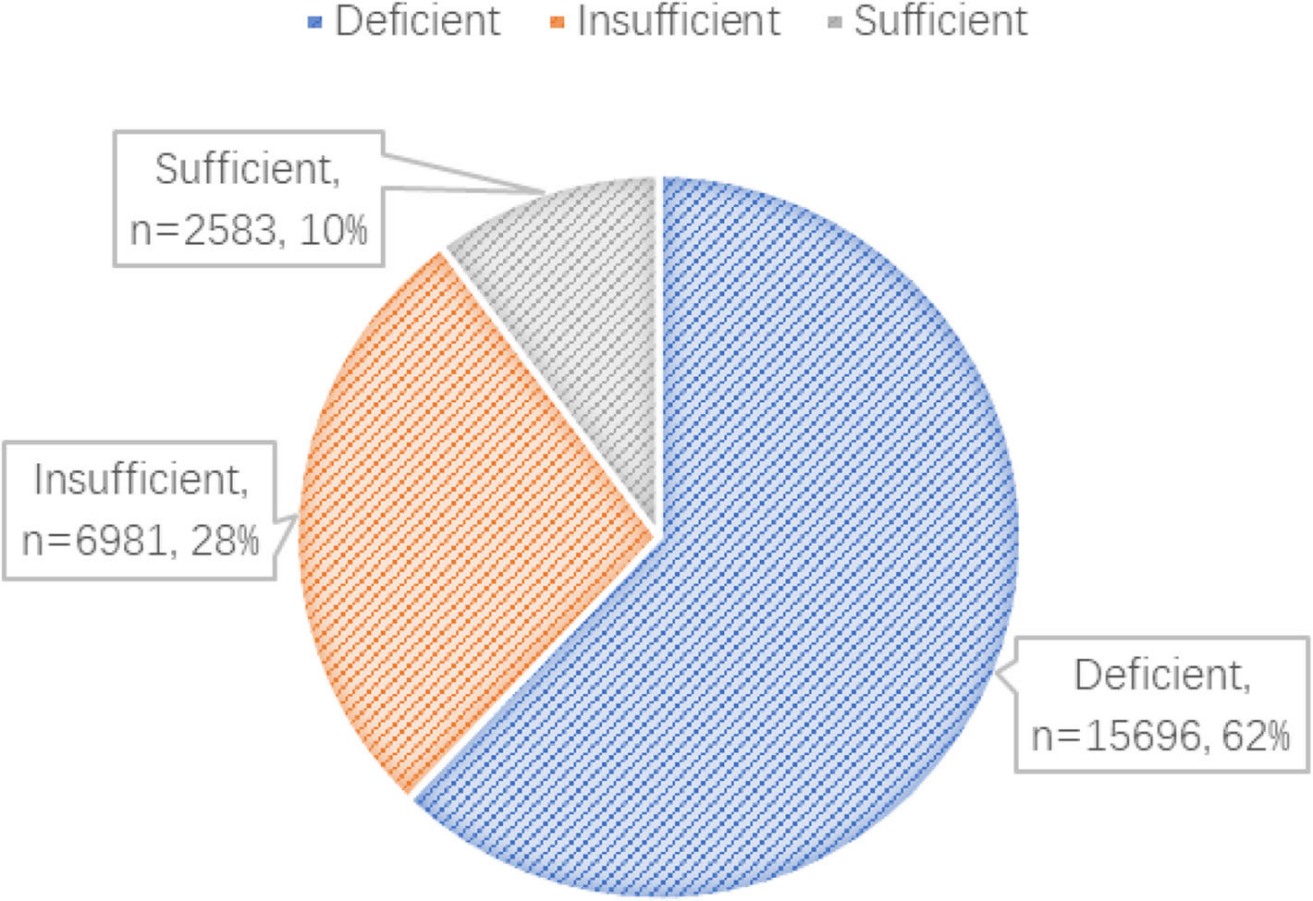
Maternal Vitamin D status in the first trimester of pregnancy

Totally, 36,297 patients were collected. After the exclusion of 894 (2.46%) women for multiple pregnancy, 5957 (16.40%) for missing medical records, 1157 (3.19%) for not performing Vitamin D test and 4895 (13.49%) not in the first trimester, results from 22,394 women were finally included in analyses (Fig. 1). The maternal serum 25(OH) D concentrations in the first trimester was 43.20 ± 0.10 nmol/L (mean ± SD) with an overall range of 2.00–124.00 nmol/L (Table 1, Fig. 2). Of the entire population, 15,696 women (67.09%) were 25(OH) D deficient, 6981(29.84%) were insufficient and only 2583 (22.2%) had sufficient 25(OH) D levels (Fig. 3).

**Table 1:** Distribution of maternal Vitamin D status in the first trimester of pregnancy

Maternal 25(OH)D status											
Mean(95%CI)	Middle	SE	Min	Max	5th	10th	25th	50th	75th	90th	95th
43.17 ± .10	42.00	15.81	2.00	124.00	20.20	23.60	30.80	42.00	53.70	64.30	70.70

### Clinical characteristics

The maternal 25(OH)D levels varied with age, pre-pregnancy BMI, season when blood was collection, number of previous pregnancy while no interaction was found in the mode of birth, and family history of diabetes or thyroid disease. Women with older age, higher pre-pregnancy BMI(*P* < 0.001) and less previous pregnancy times(*P* = .007) indicate a worse 25(OH)D status. In consistent with seasonal exposure of ultraviolet rays, concentration of vitamin D fluctuated along with recorded season, with the lowest in winter (39.40 ± 15. 60 nmol/L) and the highest in summer (47.10 ± 15.20 nmol/L), all were lower than 50 nmol/L (Table 2).

**Table 2:** Distribution of maternal demographic characteristics (*N* = 23,394)

Maternal Characteristic	25(OH)D status (nmol/L)				χ^**2**^/H ***P***- value
	< 50 (***N*** = 15,696)	50–75 (***N*** = 6981)	> 75 (***N*** = 717)	Total (N = 23,394)	
**Maternal Age(years)**					59.154 <.001^*^
<24^b^	456(2.90)	178(2.50)	8(1.10)	642(2.70)	
25-29^a^	6894(43.90)	2807(40.20)	286(39.90)	9987(42.70)	
30-34^ab^	6319(40.30)	2928(41.90)	294(41.00)	9541(40.80)	
≥35^ac^	2027(12.90)	1068(15.30)	129(18.00)	3224(13.80)	
**Pre-pregnancy BMI** (kg/m^2^)					141.771 <.001^*^
< 18.5^abc^	1932(12.30)	1037(14.90)	170(23.70)	3139(13.40)	
18.5–23.9^bc^	11,448(72.90)	5142(73.70)	493(68.80)	17,083(73.00)	
≥24^abc^	2316(14.80)	802(11.50)	54(7.50)	3172(13.60)	
**Season of blood collection**					397.256 <.001^*^
Spring	4087(26.00)	1620(23.20)	171(23.8)	5878(25.10)	
Summer	3268(20.80)	2055(29.40)	229(31.9)	5552(23.70)	
Autumn	3835(24.40)	1928(27.60)	221(29.4)	5974(25.50)	
Winter	4506(28.70)	1378(19.70)	106(14.8)	5990(25.60)	
**No. of previous pregnancies**					14.132 .007^*^
0	10,251(63.50)	4433(63.50)	451(62.90)	15,135(64.70)	
1	3817(24.30)	1732(24.80)	173(24.10)	5722(24.50)	
2	1628(10.40)	816(11.70)	93(13.00)	2537(10.80)	
**Male fetus**	8033(51.20)	3649(52.30)	381(53.10)	12,306(51.60)	3.040 .219*
**Family history of diabetes**	1314(8.40)	579(8.30)	56(7.80)	1914(8.33)	.301 .860^*^
**Family history of thyroid disease**	3	7	0	–	–
**Maternal Age(years) (Mean ± SD)**^**ab**^	30.30 ± 3.64	30.64 ± 3.70	30.93 ± 3.69	30.42 ± 3.66	58.510 <.001^#^
**Pre-pregnancy BMI** (kg/m^2^) (Mean ± SD)^**abc**^	21.23 ± 2.77	20.87 ± 2.62	20.15 ± 2.25	21.09 ± 2.72	58.643 <.001^#^

* chi-square test; # Kruskal—Wallis *H*-test; Multiple comparison were adjusted with Bonferroni calculation

a. Indicates significant differences between deficient group (< 50 nmol/L) and insufficient group(50 – 75 nmol/L);

b. Indicates significant differences between deficient group and sufficient group(> 75 nmol/L);

c. Indicates significant differences between insufficient group and sufficient group;

### Maternal outcomes

Table **3** summarized the maternal outcomes of the population. Interestingly, Women diagnosed as vitamin D insufficiency had a higher incidence rate of gestational diabetes compared with vitamin D deficiency (11.90% vs 10.70%, *P*_bonferroni_ = .020). The incidence rate of intrauterine infection, preeclampsia were different among groups but not significant after multiple comparison correction. No associations were found between gestational age (both category and numeric values), cesarean section rate, premature rupture of membranes, intrahepatic cholestasis and 2-h postpartum hemorrhage.

**Table 3:** Maternal outcomes stratified by maternal vitamin D status in the first trimester

Maternal Outcomes	25(OH)D status(nmol/L)				χ^**2**^/F/H ***P***- value
	< 50 (N = 15,696)	50–74.9 (N = 6981)	≥75 (N = 717)	Total (N = 23,394)	
**Gestational Age (weeks) (Mean±SD)**^**a**^	38.9 ± 1.4	38.9 ± 1.4	38.8 ± 1.2	38.9 ± 1.4	3.327 .036^&^
**Gestational Age(weeks) (Category)**					8.023 .431^*^
< 28	3	0	0	3	
28–31 + 6	54(0.3)	26(0.4)	0	80(0.3)	
32–33 + 6	101(0.6)	31(0.4)	4(0.6)	136(0.6)	
34–36 + 6	692(4.4)	324(4.6)	32(4.5)	1048(4.5)	
≥37	14,846(94.6)	6600(94.5)	681(95.0)	22,127(94.6)	
**Cesarean section**	6715(42.8)	3028(43.4)	301(42.0)	10,044(42.9)	0.969 .616*
**Premature rupture of membranes**	18(0.1)	12(0.2)	0	30(0.1)	2.185 .335*
**Gestational diabetes**^**a**^	1680(10.7)	831(11.9)	73(10.2)	2584(11.0)	7.648 .022*
**Preeclampsia**	440(1.9)	162(2.3)	11(1.5)	613(2.6)	7.832 .020*
**Intrahepatic cholestasis**	108(0.7)	38(0.5)	8(1.1)	154(0.7)	3.894 .143*
**Intrauterine infection**	330(2.1)	115(1.6)	10(1.4)	455(1.9)	6.422 .040*
**2-h postpartum hemorrhage(ml)**	256 ± 149	255 ± 106	244 ± 84	255 ± 137	.001 .797^#^

* chi-square test; # Kruskal—Wallis *H*-test; & ANOVA. Multiple comparison was adjusted with Bonferroni calculation

a. Indicates significant differences between deficient group (< 50 nmol/L) and insufficient group(50 – 75 nmol/L);

b. Indicates significant differences between deficient group and sufficient group(> 75 nmol/L);

c. Indicates significant differences between insufficient group and sufficient group;

### Neonatal outcomes

Most importantly, newborns delivered by women with deficient vitamin D status had a higher incidence rate of admission to NICU (Deficiency: 12.20% vs Insufficiency: 10.90% vs Sufficiency: 11.70%, *P*_bonferroni_ = .002) and a longer stay (Deficiency: 6.20 ± 4.10 vs Insufficiency:5.90 ± 3.10 vs Sufficiency: 5.10 ± 2.10, *P*_bonferroni_ = .010). Meanwhile, no correlation was observed between maternal vitamin D status and the birth weight, birth height and other outcomes. (Table 4).

**Table 4:** Neonatal outcomes stratified by maternal vitamin D status in the first trimester

Neonatal Outcomes	Maternal 25(OH)D status(nmol/L)				χ^**2**^/F/H ***P***-value
	< 50 (N = 15,696)	50–75 (N = 6981)	> 75 (N = 717)	Total (N = 23,394)	
**Birth weight (Mean ± SD)**	3346 ± 448	3340 ± 437	3329 ± 412	3343 ± 443	.833 .435^&^
Birth weight z score (Mean ± SD)	.005 ± 1.010	−.008 ± .985	−.032 ± .928	–	
Low birth weight: < 2500 g	372(2.4)	141(2.0)	14(2.0)	527(2.3)	2.995 .224^*^
Very low birth weight: < 1500 g	42(0.3)	20(0.3)	1(0.1)	63(0.3)	0.529 .768^*^
Small for gestational age: <10th percentile (3 case excluded)	322(2.1)	135(1.9)	15(2.1)	472(2.0)	1.833 .766^*^
High birth weight: > 4000 g	955(6.1)	390(5.6)	38(5.3)	1383(5.9)	2.650 .266^*^
Large for gestational age: >90th percentile	3679(23.4)	1612(23.1)	160(22.3)	5451(23.3)	2.212 .697^*^
**Birth length (Mean ± SD)**	49.9 ± 1.4	49.8 ± 1.4	49.9 ± 1.1	49.9 ± 1.4	.689 .502^&^
**Apgar score (Mean ± SD)**	9.7 ± .9	9.7 ± 1.0	9.7 ± .8	9.7 ± .9	1.938 .144^&^
**Apgar score**					7.144 .308^*^
0–3	28(.2)	17(.2)	0	45(.2)	–
4–6	106(.7)	43(.6)	2(.3)	151(.6)	
7–8	270(1.7)	137(2.0)	9(1.3)	416(1.8)	
9–10	15,292(97.4)	6784(97.2)	706(98.5)	22,782(97.4)	
**Admission to neonatal intensive care unit**	1918(12.2)	761(10.9)	67(9.3)	2746(11.7)	12.200 .002^*^
**Admission to neonatal intensive care unit (Mean ± SD)**	6.2 ± 4.1	5.9 ± 3.1	5.1 ± 2.1	6.1 ± 3.2	.010^#^
**Any respiratory disorders**^**¶**^	334(2.1)	131(1.9)	16(2.2)	481(2.1)	1.205 .300^*^
Dyspnea (clinical diagnosis)	8(0.1)	5(0.1)	0	13(0.1)	.782 .676^*^
Wet lung	266(1.7)	107(1.5)	13(1.8)	386(1.6)	.902 .637^*^
Aspiration pneumonia	5	3	0	8	–
Pulmonary arterial hypertension	2	0	0	2	–
Pneumothorax	30(0.2)	6(0.1)	1(0.1)	37(0.2)	3.402 .183^*^
Bronchopulmonary dysplasia	2	0	0	2	–
**Neonatal convulsion**	6	6	1	13	–
**Hyperbilirubinemia**	971(6.2)	434(6.2)	30(4.2)	1435(6.1)	4.487 .087^*^
**Necrotizing enterocolitis**	79(0.5)	26(0.4)	4(0.6)	109(0.5)	1.919 .383^*^
**Sepsis**	29(0.2)	14(0.2)	2(0.3)	45(0.2)	–
**Retinopathy of prematurity**	19	7	0	26	–
**Neonatal death**	1	1	0	2	–

* chi-square test; # Kruskal—Wallis *H*-test; & ANOVA. Bonferroni adjustment was performed

Multiple comparisons were adjusted with Bonferroni calculation. **¶** any respiratory disorders (including dyspnea, wet lung, aspiration pneumonia, pulmonary arterial hypertension, pneumothorax, bronchopulmonary dysplasia);

a. Indicates significant differences between deficient group (< 50 nmol/L) and insufficient group(50 – 75 nmol/L);

b. Indicates significant differences between deficient group and sufficient group(> 75 nmol/L);

c. Indicates significant differences between insufficient group and sufficient group;

### Unadjusted and adjusted risk factors analysis

**Fig. 4:**
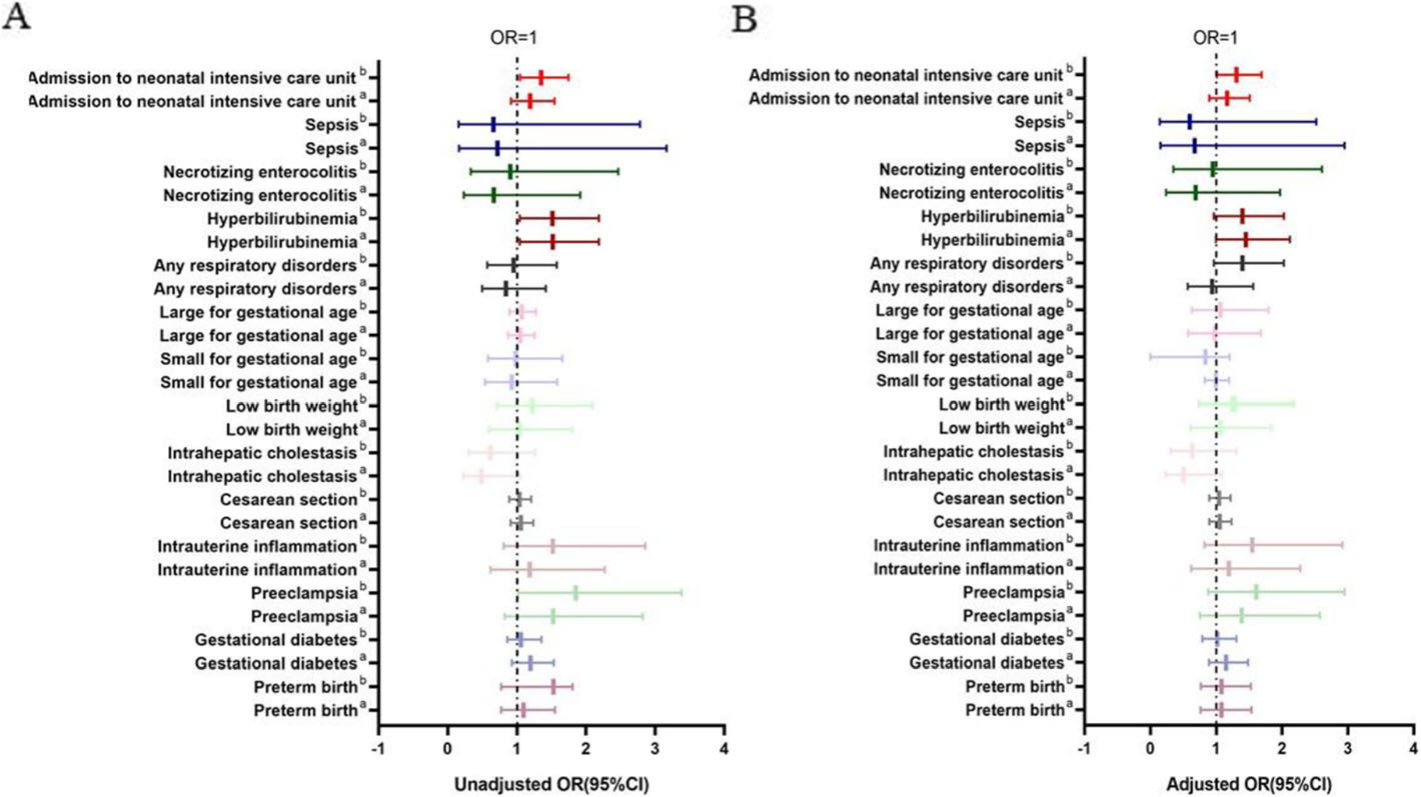
The Forest Plot of unasjusted and adjusted models. A. The unadjusted model. B. The adjusted model (Adjusted for maternal age (category variable), pre-pregnancy BMI (category variable), fetus sex, collection season of blood sample, No. of previous pregnancies. Using vitamin D sufficiency (> 75 nmol/L) as a reference. a. Insufficient group vs sufficient group. b. Deficient group vs sufficient group. The dot line indicates where OR = 1

Then we burrowed deep into some common complications of mothers and newborns which consist of preterm birth, gestational diabetes, preeclampsia, intrauterine inflammation, cesarean section, premature rupture of membrane, intrahepatic cholestasis for mothers and low birth weight, small for gestational age, large for gestational age, admission to NICU hospitalization, hyperbilirubinemia, necrotizing enterocolitis, sepsis for newborns (Table 5, Fig. 4).

**Table 5:** Unadjusted and adjusted odds ratios for maternal and neonatal stratified by vitamin D status

	Unadjusted OR (95% CI) and ***P*** Value			Adjusted OR^**a**^ (95% CI) And ***P*** Value		
Maternal outcomes	≥75	50–74.9	< 50	≥75	50–74.9	< 50
**Preterm birth**	1.00 -	1.092(.769–1.551) .623	1.086(.771–1.529) .637	1.00 -	1.080(.760–1.535) .667	1.078(.765–1.520) .667
**Gestational diabetes**	1.00 -	1.192(.926–1.535) .173	1.057(.826–1.354) .658	1.00 -	1.145(.886–1.478) .300	1.014(.789–1.303) .915
**Preeclampsia**	1.00 -	1.525(.824–2.822) .179	1.851(1.013–3.383) .045	1.00 -	1.387(.748–2.571) .299	1.609(.879–2.948) .123
**Intrauterine inflammation**	1.00 -	1.184(.618–2.270) .611	1.518(.806–2.861) .196	1.00 -	1.187(.618–2.278) .607	1.545(.817–2.920) .181
**Cesarean section**	1.00 -	1.059(.906–1.237) .437	1.033(.888–1.203) .672	1.00 -	1.049(.895–1.230) .556	1.040(.890–1.214) .622
**Intrahepatic cholestasis**	1.00 -	.485(.225–1.044) .064	.614(.298–1.264) .186	1.00 -	.501(.232–1.079) .078	.633(.306–1.308) .217
**Neonatal outcomes**						
**Low birth weight**	1.00 -	1.035(.594–1.803) .903	1.219(.711–2.090) .471	1.00 -	1.055(.606–1.839) .849	1.263(.736–2.169) .397
**Small for gestational age**	1.00 -	.923(.538–1.582) .770	.980(.581–1.654) .941	1.00 -	.991(.822–1.196) .927	.999(.832–1.199) .989
**Large for gestational age**	1.00 -	1.045(.869–1.257) .638	1.066(.891–1.276) .485	1.00 -	.978(.569–1.678) .935	1.060(.626–1.793) .829
**Admission to neonatal intensive care unit**	1.00 -	1.187(.913–1.543) .201	1.350(1.045–1.744) .022	1.00 -	1.160(.892–1.509) .269	1.305(1.010–1.687) .042
**Any respiratory disorders**	1.00 -	.838(.496–1.416) .509	.950(.572–1.578) .842	1.00 -	.824(.487–1.394) .470	.937(.563–1.559) .803
**Hyperbilirubinemia**	1.00 -	1.518(1.042–2.189) .031	1.510(1.042–2.189) .030	1.00 -	1.451(.993–2.118) .054	1.397(.963–2.027) .079
**Necrotizing enterocolitis**	1.00 -	.666(.232–1.915) .451	.902(.329–2.469) .840	1.00 -	.684(.238–1.969) .482	.947(.345–2.603) .916
**Sepsis**	1.00 -	.718(.163–3.167) .662	.662(.158–2.779) .662	1.00 -	.668(.151–2.951) .594	.597(.141–2.523) .483

^a^Adjusted for maternal age (category variable), pre-pregnancy BMI (category variable), fetus sex, collection season of blood sample, No. of previous pregnancies. Using vitamin D sufficiency (> 75 nmol/L) as a reference

Interestingly, maternal vitamin D deficiency was a dependent risk factor for admission to NICU (unadjusted OR = 1.350, 95%CI (1.045–1.744), *P* =.022; adjusted OR = 1.305, 95%CI (1.010–1.687), *P* = .042). To determine the potential confounding factor, we further analyzed demographic baseline of mothers and neonatal outcomes between newborns whether to be admitted to NICU (Table 6). The results indicated that women whose infants were transferred to NICU after delivery had a slightly lower vitamin D concentration (42.32 ± 15.18 nmol/L vs 43.28 ± 15.89, *P* = .010). Furthermore, lower maternal age (30.20 ± 3.50 vs 30.50 ± 3.70, *P* =.006), higher pre-pregnancy BMI (21.10 ± 3.40 vs 20.80 ± 3.60, *P* ≤ .001) and gestational age at birth (39.00 ± 1.20 vs 38.20 ± 2.40, *P* = .001) was observed in NICU group. NICU group had a lower cesarean section rate (38.90% vs 43.50%, *P* ≤ .001), Apgar score (9.70 ± 0.90 vs 9.90 ± .59, *P* < .001), birth weight (3172.00 ± 619.00 vs 3366.00 ± 409.00 *P ≤* .001), and birth length(49.20 ± 2.40 vs 49.90 ± 1.10, *P ≤* .001).

**Table 6:** Characteristics of newborns admitted to NICU (*N* = 2746)

Maternal characteristics and Pregnant outcomes	Admission to neonatal intensive care unit		χ^**2**^/Z/ ***P***- value
	Without (***N*** = 20,648)	With (N = 2746)	
**25(OH)D levels (Mean ± SD)**	43.283 ± 15.893	42.319 ± 15.183	−2.590 .010^&^
**25(OH)D levels (N%)**			12.200 .002^*^
<50^a^	13,778 (66.7)	1918 (69.8)	
50–74.9^a^	6220 (30.1)	761 (27.7)	
≥75^a^	650 (3.1)	67(2.4)	
**Maternal Age(years)** **(Mean ± SD)**	30.5 ± 3.7	30.2 ± 3.5	−2.759 .006^&^
**Maternal Age(years) (N(%))**			17.364 .001^*^
<24^a^	628 (2.8)	14 (2.1)	
25-29^a^	9775 (42.4)	212 (44.9)	
30–34	9351 (40.7)	244 (41.2)	
≥35^a^	3165 (14.1)	114 (11.7)	
**Pre-pregnancy BMI (kg/m2)** **(Mean ± SD)**	20.8 ± 3.6	21.1 ± 3.4	20.731 <.001^#^
**Pre-pregnancy BMI (kg/m) (N(%))**			
< 18.5^a^	2813 (13.6)	326 (11.9)	
18.5–23.9	15,105 (73.2)	1978 (72.0)	
≥24^a^	2730(13.2)	442 (16.1)	
**Season of blood collection**			8.109 .044^*^
Spring ^a^	5132(24.9)	746 (27.2)	
Summer	4910 (23.8)	642 (23.4)	
Autumn	5314 (25.7)	660 (24.0)	
Winter	5292(25.6)	698 (25.4)	
**No. of previous pregnancies**			4.758 .093^*^
0^a^	13,311 (64.5)	1824 (66.4)	
1	5072 (24.6)	650 (23.7)	
2+	2265 (11.0)	272 (9.9)	
**Gestational age at birth, weeks** **(Mean ± SD)**	39.0 ± 1.2	38.2 ± 2.4	−10.445 <.001^&^
**Gestational age at birth (N(%))**			1976.277 <.001^*^
< 28	2	1	
28–31 + 6	32 (.2)	48 (1.7)	
32–33 + 6	13 (.1)	123 (4.5)	
34–36 + 6	614 (3.0)	434 (15.8)	
≥37	19,987 (96.8)	2140 (83.5)	
**Male (N%)**	10,600 (51.3)	1463 (53.3)	3.655 .056^*^
**Cesarean section (N%)**	8975 (43.5)	1069 (38.9)	22.366 <.001^*^
**2-h postpartum hemorrhage(ml)**	241 ± 155	255 ± 137	−7.964 <.001^&^
**Apgar score(Mean ± SD)**	9.9 ± .6	9.7 ± .9	−18.128 <.001^&^
**Apgar score** **(N%)**			233.297 <.001^*^
0–3	37(.2)	8(.3)	
4-6^a^	97(.5)	54(2.0)	
7-8^a^	289(1.4)	127(4.6)	
9-10^a^	20,225(98.0)	2557(93.1)	
**Neonatal birth weight(g)**	3366 ± 409	3172 ± 619	−12.208 <.001^&^
**Neonatal birth height(cm)**	49.9 ± 1.1	49.2 ± 2.4	−12.976 <.001^&^

* chi-square test; # Student’s t test;& Mann-Whitney U test. Multiple comparison was adjusted with Bonferroni calculation

a. The difference was significant (*P* < 0.05) after multiple comparison correction

The results also showed that women diagnosed as vitamin D deficiency had a higher risk for preeclampsia (OR = 1.851, 95%CI (1.013–3.383), *P* = .045). However, the trend was not observed after adjusting for maternal age (category variable), pre-pregnancy BMI (category variable), fetus sex, season of blood sample collection and No. of previous pregnancies. Besides, newborn delivered by vitamin D deficient women were apt to develop hyperbilirubinemia (unadjusted OR = 1.350, CI (1.045–1.744), *P* = .022; adjusted OR = 1.397, 95%CI (.963–2.027), *P* = .079) as well as insufficient group (unadjusted OR = 1.518, 95%CI (1.042–2.189), *P* = .031; adjusted OR = 1.451, 95%CI (.993–2.118), *P* = .054).

## Discussion

In the study, we investigated the maternal vitamin D status in the first trimester of pregnancy and the relations between vitamin D concentration and maternal as well as neonatal outcomes.

### The prevalence of vitamin D deficiency and its risk factors

In spite of its importance, the vitamin D status is not optimized among population especially pregnant women who in great amount need of it. Vitamin D deficiency (VDD), defined as serum 25(OH)D concentration < 50.00 nmol/L) [11], is prevalent from equatorial areas to Northern Europe, ranging from 26 to 95% [12–15]. The same trend was observed in our study since 67.09% women were diagnosed as VDD. Compared with other districts in China, with 90.2% in Beijing (39.9°N) [16], 83.6% in Guiyang (27.2°N) [17], 18.9% in Guangzhou (23°N) [18]. Geographic position, dietary structure, character of job might contribute to the disparity. Why does VDD happen so frequently? Previous researches have unveiled that vitamin D can be obtained from diverse plant and animal dietary sources as well as sunlight exposure. Then, all sources of vitamin D are transformed into 25 hydroxyvitamin D, the predominant but inactive circulating form of vitamin D, in the liver by 25-hydroxylases. CYP27B1(1-alpha-hydroxylase) in the kidney mainly and other sites including placenta and brain convert 25(OH)D into 1,25(OH)_2_D [19, 20] which induce both genomic and non-genomic effects mediated by VDR [21, 22]. Considered the comprehensive metabolic pathways, dietary depletion, seasonal sunlight exposure lacking, adiposity and genetic variants contribute to the incidence of VDD [23]. For instance, the negative correlation between vitamin D status and BMI in the study could be explained by a relatively smaller skin surface for vitamin D synthesis [24]. In addition, since vitamin D is a fat-soluble molecular, it might be stored in fat tissue instead of being detecting as free form. According to Chen, each additional unit (1 kg/m^2^) of pre-pregnancy BMI indicated a 0.23 ng/mL increase in 25(OH)D concentration [25]. Interestingly, in our study, the level of Vitamin D increased with maternal age and the number of previous pregnancies. It may due to that multipara had experience in nutrient supplements, lifestyle changes and healthcare services requirements than novice mothers.

### Maternal and neonatal outcomes

Not only does maternal VDD associates with increased risk of gestational diabetes and pre-eclampsia [26, 27], but also directly effects on offspring health as low birth weight, impaired brain development, obesity and insulin resistance [28–30].

### *Admission to NICU*

Of the most important, we found the incidence of newborns admitted to NICU was strongly associated with maternal vitamin D status in the first trimester of pregnancy. When compared with women with sufficient serum vitamin D concentration (≥ 75 nmol/L), women diagnosed as insufficiency (50–74.9 nmol/L) and deficiency (< 50 nmol/L) had higher risk of delivering babies admitted to NICU in both unadjusted and adjusted models (Deficiency: unadjusted OR = 1.350, 95%CI (1.045–1.744), *P* = .022, adjusted OR = 1.305, 95% CI (1.010–1.687), *P* < 0.001). Meanwhile, the result revealed a trend that the risk of NICU hospitalization of newborns increased as the maternal vitamin D status deteriorated. Newborns admitted to NICU were premature or companied with severe complications such as septicemia, hypoxic-ischemic encephalopathy (HIE), necrotizing enterocolitis [31]. In the one hand, some disease may have long-term sequela (malformation, Neurodevelopmental abnormality). However, newborns might be transferred to NICU for diverse reasons, it can be a practical indicator for both prognostic and economic consequences. Neonatal intensive care (NIC) cost 26.2 billion USD a year in the United States [32]. With escalating health expenditure, resource allocation by the government or public sector will be determined by health economic evaluations of new technologies or innovations. Since vitamin D supplementation was convenient and effective to reduce the incidence of NICU hospitalization, our research provided a practical recommendation for decision-makers. What needs to be confirmed is how to intervene, for vitamin D may plays different roles across the pregnancy, from placenta implantation to fetal bone formation.

### Preterm birth*Preterm birth*

Globally, 11% of newborns are preterm birth, leading to 15 million premature infants. The incidence of preterm birth is increasing in many countries, meanwhile, the survival rate of preterm babies has dramatically improved in developed countries [33, 34]. Preterm birth represents a significant cause of death and can lead to serious harm to survivors all around the world [35]. In our study, maternal vitamin D status had no correlation with preterm birth. Nonetheless, a possible negative correlation between maternal vitamin D status and preterm birth was reported [36, 37]. A recent meta-analysis consisted of 24 observational studies revealed the association between low vitamin D levels (< 50.00 nmol/L) and increased risk of preterm birth (OR = 1.58, 95%CI (1.08 to 2.31) [38–41] while studies from New Zealand [42] and China [43] shared the similar view with us. The discrepancy may due to different sample size, research methods confounding factors, various 25(OH)D cutoff values, population characteristics and methods for measuring vitamin D status. Since we haven’t excluded some cases with risk factors related to PTB, the certain relation might be concealed. In addition, molecular research demonstrated that vitamin D metabolism can be affected by single nucleotide polymorphisms (SNP) of VDR genes such as BsmI, FokI, TaqI, and ApaI, resulting in different maternal serum vitamin D concentrations and functions in downstream, even given the same amount of supplement [44].

### *Gestational diabetes mellitus*

GDM, manifests as insulin resistance, increased inflammatory factors and oxidative stress [45], can result in adverse maternal outcomes and long-term sequelae in the offspring [46]. A RCT in Iran investigated that vitamin D supplementation in high-risk pregnancies women leaded to a significant reduction in fasting plasma glucose concentration, insulin levels and HOMA-IR accompanied with lower serum LDL-cholesterol and total cholesterol [7]. However, a meta-analysis including several RCTs found no beneficial effect of vitamin D supplementation on indicators of glucose homeostasis such as fasting plasma glucose (FPG), insulin, HbA1cand lipo-metabolism spectrum [47]. In this study, we found the correlation between maternal vitamin D status and GDM was not noted in unadjusted and adjusted models. Researches revealed vitamin D can intervene glucose homeostasis from different layers: immune cell infiltration among glandular cells results in inflammation and functional pancreatic alteration. As an anti-inflammatory molecular, vitamin D can rebuild insulin secretion function to some extent [48]. Through indirect way, vitamin D increase duodenal absorption and renal resorption of calcium, a vital downstream factor of insulin pathway [49]. Moreover, vitamin D receptors (VDRs) were found participating in promoting insulin sensitivity [50]. For clinician and policymakers, the role of vitamin D supplementation in gestational diabetes does not come into light. [51]. Fortunately, some double-blind RCTs indicated beneficial effect of vitamin D during the first and second trimesters of pregnancy supplementation on GDM [23].

### *Preeclampsia*

In the study, Preeclampsia was not an independent risk factor whether adjusted for confounding factors. In consistent with our study, few observational studies showed negative results for the effects of vitamin D on preeclampsia [52]. However, a meta-analysis consists of 12 studies indicated that women with low maternal serum 25(OH)D concentrations were susceptible to preeclampsia. Although the poor quality of evidence raised vagueness in causality [53]. The same results were reported in another recently updated meta-analysis including 23 studies with slightly increased fixed (1.33) [54]. The disagreement of these researches may result from different time when the blood samples were collected, since vitamin D has diverse roles throughout the whole pregnancy including the regulation of trophoblast differentiation and EVT invasion of the decidua and myometrium at early stage [55].

### *Intrauterine inflammation*

Intrauterine inflammation is often related to chorioamnionitis, a common cause of preterm birth leading to adverse neonatal outcomes [56, 57]. If not treated timely and properly, long-term outcomes as neurodevelopmental sequelae and chronic lung disease might influence life quality in adulthood [58, 59]. Chorioamnionitis may be classified as clinical chorioamnionitis and subclinical/histologic chorioamnionitis based on clinical signs and laboratory evidences. A recent observational study suggested that vitamin D in early pregnancy was a protective factor for intrauterine infection and neonatal sepsis was associated since it reduced placental inflammation [14]. According to our results, no correlation was found between intrauterine inflammation and vitamin D. It might be due to the reason that we confirmed the diagnosis based on clinical signs other than histological evidence. Besides, the etiology of chorioamnionitis is heterogeneous, such as bacterial or virus infection from vagina or blood. Even chronic stress could lead to it [60]. In animal research, when exposed to lipopolysaccharide (LPS), deficient vitamin D diet mice were observed with an elevation mRNA for Il-6, IFN-γ, TNF-α, classical inflammatory factors, in placenta. However, researchers haven’t reached agreement on the anti-inflammatory effect of vitamin D during pregnancy.

### *Low birth weight/SGA*

Consistent with our study, some researchers noted maternal serum vitamin D were scarcely related to SGA, while others showed that mothers with lower vitamin D levels were apt to giving birth to SGA fetuses [4]. Wang indicated that maternal VDD might be an independent risk factor for poor fetal growth, for each 1 ng/ml decrease of 25(OH)D accompanied by 19% increase of the risk of SGA [61]. This might be explained by the following mechanisms: First, maternal VDD may affect directly fetal bone metabolism. Then, as a member of steroid hormone family, vitamin D could interact with other hormones vital for fetal development, such as thyroid hormone [62]. The homeostasis of metabolism of nutrient in pregnancy could be modulated by vitamin D, affecting fetal development [63].

### Strengths of the study

This study with large sample size and focused on the vitamin D status of pregnant women in the first trimester in easter coastal China, Shanghai. The result highlights the deficiency of vitamin D was prevalent, not only in developed countries. We analyzed a potential correlation between vitamin D deficiency and maternal and neonatal outcomes such as preterm birth, gestational diabetes mellitus, preeclampsia, intrauterine inflammation, SGA and admission to NICU of newborns. Of the most important, we found a strong association between maternal vitamin D levels with NICU hospitalization, a meaningful indicator of long-term health of newborns and provided policy-makers with a supportive evidence to put emphasis on vitamin D supplementation in early period of pregnancy.

### Limitations and weaknesses of the study

First, this study of its retrospective nature, we couldn’t determine whether lower maternal vitamin D concentrations would lead to a higher risk of NICU admission rate. Secondly, some covariates such as dietary, lifestyle, sunshine exposure, clothing preferences and extra supplementation of vitamin D were not available in the study. Finally, as every coin has two sides, we didn’t record the vitamin D levels of the second and third trimesters, just focused on the first trimester. It might be possible that the levels of vitamin D would have changed progressively. Further well-designed and prospective researches are necessary for clarify the causal link between maternal vitamin D status and the outcomes of mothers and newborns.

## Conclusion

Maternal vitamin D deficiency (25(OH)D < 50 nmol/L) was prevalent in easter coastal China. The incidence rate of GDM as well as preeclampsia was higher in vitamin D insufficient group while vitamin D deficiency group was liable to intrauterine infection. Furthermore, low vitamin D status in pregnant women was an independent risk factor for admission to NICU. More well-designed perspective researches are necessary to clarify the role of vitamin D in the early stage of pregnancy.

## Data Availability

The data used during the study are available from the corresponding author on reasonable request.

## Abbreviations

DOHaD: Developmental Origin of Health and Disease
VDD: Vitamin D deficiency
BMI: Body Mass Index
NICU: Neonatal Intensive Care Unit
PTB: Preterm Birth
PROM: Preterm Rupture of Membranes
GDM: Gestational Diabetes Mellitus
LBG: Low birth weight
VLBW: Very low birth weight
SGA: Small for gestational age
LGA: Large for gestational age
LPS: Lipopolysaccharide

## References

[1] Wacker M, Holick MF (2013). Vitamin D - effects on skeletal and extraskeletal health and the need for supplementation. Nutrients..

[2] Wilson LR, Tripkovic L, Hart KH, Lanham-New SA (2017). Vitamin D deficiency as a public health issue: using vitamin D2 or vitamin D3 in future fortification strategies. Proc Nutr Soc.

[3] Palacios C, Gonzalez L. Is vitamin D deficiency a major global public health problem? J Steroid Biochem Mol Biol. 2014; 144 Pt A:138–145. 10.1016/j.jsbmb.2013.11.003 PMC4018438 24239505

[4] Aghajafari F, Nagulesapillai T, Ronksley PE, Tough SC, O'Beirne M, Rabi DM (2013). Association between maternal serum 25-hydroxyvitamin D level and pregnancy and neonatal outcomes: systematic review and meta-analysis of observational studies. BMJ..

[5] Dawodu A, Saadi HF, Bekdache G, Javed Y, Altaye M, Hollis BW (2013). Randomized controlled trial (RCT) of vitamin D supplementation in pregnancy in a population with endemic vitamin D deficiency. J Clin Endocrinol Metab.

[6] Diogenes MEL, Bezerra FF, Rezende EP, Taveira MF, Pinhal I, Donangelo CM (2013). Effect of calcium plus vitamin D supplementation during pregnancy in Brazilian adolescent mothers: a randomized, placebo-controlled trial. Am J Clin Nutr.

[7] Asemi Z, Tabassi Z, Heidarzadeh Z, Khorammian H, Sabihi S-S, Samimi M (2012). Effect of calcium-vitamin D supplementation on metabolic profiles in pregnant women at risk for pre-eclampsia: a randomized placebo-controlled trial. Pak J Biol Sci.

[8] Rosendahl J, Pelkonen AS, Helve O, Hauta-Alus H, Holmlund-Suila E, Valkama S, et al. High-dose vitamin D supplementation does not prevent allergic sensitization of infants. J Pediatr. 2019;209:139-145.e1. 10.1016/j.jpeds.2019.02.021 30902420

[9] McGrath J (2001). Does 'imprinting' with low prenatal vitamin D contribute to the risk of various adult disorders?. Med Hypotheses.

[10] Ponsonby A-L, Lucas RM, Lewis S, Halliday J (2010). Vitamin D status during pregnancy and aspects of offspring health. Nutrients..

[11] Holick MF, Binkley NC, Bischoff-Ferrari HA, Gordon CM, Hanley DA, Heaney RP, Murad MH, Weaver CM (2011). Evaluation, treatment, and prevention of vitamin D deficiency: an Endocrine Society clinical practice guideline. J Clin Endocrinol Metab.

[12] Christoph P, Challande P, Raio L, Surbek D (2020). High prevalence of severe vitamin D deficiency during the first trimester in pregnant women in Switzerland and its potential contributions to adverse outcomes in the pregnancy. Swiss Med Wkly.

[13] Toko EN, Sumba OP, Daud II, Ogolla S, Majiwa M, Krisher JT, et al. Maternal vitamin D status and adverse birth outcomes in children from rural Western Kenya. Nutrients. 2016;8(12):794. 10.3390/nu8120794 PMC5188449 27941597

[14] Zhang Q, Chen H, Wang Y, Zhang C, Tang Z, Li H (2019). Severe vitamin D deficiency in the first trimester is associated with placental inflammation in high-risk singleton pregnancy. Clin Nutr.

[15] Nimitphong H, Holick MF (2013). Vitamin D status and sun exposure in Southeast Asia. Dermatoendocrinol..

[16] Song SJ, Si S, Liu J, Chen X, Zhou L, Jia G (2013). Vitamin D status in Chinese pregnant women and their newborns in Beijing and their relationships to birth size. Public Health Nutr.

[17] Xiang F, Jiang J, Li H, Yuan J, Yang R, Wang Q, Zhang Y (2013). High prevalence of vitamin D insufficiency in pregnant women working indoors and residing in Guiyang, China. J Endocrinol Invest.

[18] Zhou J, Su L, Liu M, Liu Y, Cao X, Wang Z, Xiao H (2014). Associations between 25-hydroxyvitamin D levels and pregnancy outcomes: a prospective observational study in southern China. Eur J Clin Nutr.

[19] Townsend K, Evans KN, Campbell MJ, Colston KW, Adams JS, Hewison M (2005). Biological actions of extra-renal 25-hydroxyvitamin D-1alpha-hydroxylase and implications for chemoprevention and treatment. J Steroid Biochem Mol Biol.

[20] Anderson CM, Gillespie SL, Thiele DK, Ralph JL, Ohm JE (2018). Effects of maternal vitamin D supplementation on the maternal and infant Epigenome. Breastfeed Med.

[21] Wang TJ, Zhang F, Richards JB, Kestenbaum B, van Meurs JB, Berry D (2010). Common genetic determinants of vitamin D insufficiency: a genome-wide association study. Lancet..

[22] Kennedy DA, Cooley K, Skidmore B, Fritz H, Campbell T, Seely D (2013). Vitamin d: pharmacokinetics and safety when used in conjunction with the pharmaceutical drugs used in cancer patients: a systematic review. Cancers (Basel).

[23] Ross AC (2011). The 2011 report on dietary reference intakes for calcium and vitamin D. Public Health Nutr.

[24] Lagunova Z, Porojnicu AC, Lindberg F, Hexeberg S, Moan J (2009). The dependency of vitamin D status on body mass index, gender, age and season. Anticancer Res.

[25] Chen Y-H, Fu L, Hao J-H, Wang H, Zhang C, Tao F-B, Xu D-X (2018). Influent factors of gestational vitamin D deficiency and its relation to an increased risk of preterm delivery in Chinese population. Sci Rep.

[26] Bodnar LM, Catov JM, Simhan HN, Holick MF, Powers RW, Roberts JM (2007). Maternal vitamin D deficiency increases the risk of preeclampsia. J Clin Endocrinol Metab.

[27] Brannon PM (2012). Vitamin D and adverse pregnancy outcomes: beyond bone health and growth. Proc Nutr Soc.

[28] Veena SR, Gale CR, Krishnaveni GV, Kehoe SH, Srinivasan K, Fall CH (2016). Association between maternal nutritional status in pregnancy and offspring cognitive function during childhood and adolescence; a systematic review. BMC Pregnancy Childbirth.

[29] Maia-Ceciliano TC, Dutra RR, Aguila MB, Mandarim-De-Lacerda CA (2019). The deficiency and the supplementation of vitamin D and liver: lessons of chronic fructose-rich diet in mice. J Steroid Biochem Mol Biol.

[30] Cooper C, Harvey NC, Bishop NJ, Kennedy S, Papageorghiou AT, Schoenmakers I (2016). Maternal gestational vitamin D supplementation and offspring bone health (MAVIDOS): a multicentre, double-blind, randomised placebo-controlled trial. Lancet Diabetes Endocrinol.

[31] Liao X-P, Chipenda-Dansokho S, Lewin A, Abdelouahab N, Wei S-Q (2017). Advanced neonatal medicine in China: a National Baseline Database. PLoS One.

[32] Rios JD, Shah PS, Beltempo M, Louis D, Mukerji A, Premji S, Shah V, Lee SK, Pechlivanoglou P. Costs of neonatal intensive care for Canadian infants with preterm birth. J Pediatr. 2021;229:161–167.e12. 10.1016/j.jpeds.2020.09.045 32979384

[33] Deindl P, Diemert A (2020). From structural modalities in perinatal medicine to the frequency of preterm birth. Semin Immunopathol.

[34] Liu L, Oza S, Hogan D, Perin J, Rudan I, Lawn JE, Cousens S, Mathers C, Black RE (2015). Global, regional, and national causes of child mortality in 2000-13, with projections to inform post-2015 priorities: an updated systematic analysis. Lancet..

[35] Blencowe H, Cousens S, Chou D, Oestergaard M, Say L, Moller A-B, Kinney M, Lawn J. Born too soon: the global epidemiology of 15 million preterm births. Reprod Health. 2013; 10 Suppl 1:S2. 10.1186/1742-4755-10-S1-S2 PMC3828585 24625129

[36] Dawson-Hughes B, Heaney RP, Holick MF, Lips P, Meunier PJ, Vieth R (2005). Estimates of optimal vitamin D status. Osteoporos Int.

[37] Morley R, Carlin JB, Pasco JA, Wark JD (2006). Maternal 25-hydroxyvitamin D and parathyroid hormone concentrations and offspring birth size. J Clin Endocrinol Metab.

[38] Tabatabaei N, Auger N, Herba CM, Wei S, Allard C, Fink GD, Fraser WD (2017). Maternal vitamin D insufficiency early in pregnancy is associated with increased risk of preterm birth in ethnic minority women in Canada. J Nutr.

[39] Wei S-Q, Qi H-P, Luo Z-C, Fraser WD (2013). Maternal vitamin D status and adverse pregnancy outcomes: a systematic review and meta-analysis. J Matern Fetal Neona.

[40] Flood-Nichols SK, Tinnemore D, Huang RR, Napolitano PG, Ippolito DL (2015). Vitamin D deficiency in early pregnancy. PLoS One.

[41] Hollis BW, Wagner CL (2017). Vitamin D supplementation during pregnancy: improvements in birth outcomes and complications through direct genomic alteration. Mol Cell Endocrinol.

[42] Boyle VT, Thorstensen EB, Mourath D, Jones MB, McCowan LME, Kenny LC, Baker PN (2016). The relationship between 25-hydroxyvitamin D concentration in early pregnancy and pregnancy outcomes in a large, prospective cohort. Br J Nutr.

[43] Yu L, Guo Y, Ke H-J, He Y-S, Che D, Wu J-L (2019). Vitamin D status in pregnant women in southern China and risk of preterm birth: a large-scale retrospective cohort study. Med Sci Monit.

[44] Rosenfeld T, Salem H, Altarescu G, Grisaru-Granovsky S, Tevet A, Birk R (2017). Maternal-fetal vitamin D receptor polymorphisms significantly associated with preterm birth. Arch Gynecol Obstet.

[45] Wahab RJ, Scholing JM, Gaillard R. Maternal early pregnancy dietary glycemic index and load, fetal growth, and the risk of adverse birth outcomes. Eur J Nutr. 2021;60(3):1301–11. 10.1007/s00394-020-02327-9 PMC7987612 32666314

[46] Tsai P-JS, Roberson E, Dye T (2013). Gestational diabetes and macrosomia by race/ethnicity in Hawaii. BMC Res Notes.

[47] Akbari M, Moosazaheh M, Lankarani KB, Tabrizi R, Samimi M, Karamali M, Jamilian M, Kolahdooz F, Asemi Z (2017). The effects of vitamin D supplementation on glucose metabolism and lipid profiles in patients with gestational diabetes: a systematic review and meta-analysis of randomized controlled trials. Horm Metab Res.

[48] Alvarez JA, Ashraf A (2010). Role of vitamin d in insulin secretion and insulin sensitivity for glucose homeostasis. Int J Endocrinol.

[49] Hassan-Smith Z, Hewison M, Gittoes N (2019). Vitamin D supplementation and prevention of type 2 diabetes. N Engl J Med.

[50] Vaidya A, Williams JS (2012). Vitamin D and insulin sensitivity: can gene association and pharmacogenetic studies of the vitamin D receptor provide clarity?. Metabolism..

[51] Looker AC, Gunter EW. Hypovitaminosis D in medical inpatients. N Engl J Med. 1998;339(5):344–5. 10.1056/NEJM199807303390512 9696642

[52] Davies-Tuck M, Yim C, Knight M, Hodges R, Doery JCG, Wallace E (2015). Vitamin D testing in pregnancy: does one size fit all?. Aust N Z J Obstet Gynaecol.

[53] Hyppönen E, Cavadino A, Williams D, Fraser A, Vereczkey A, Fraser WD, Bánhidy F, Lawlor D, Czeizel AE (2013). Vitamin D and pre-eclampsia: original data, systematic review and meta-analysis. Ann Nutr Metab.

[54] Akbari S, Khodadadi B, Ahmadi SAY, Abbaszadeh S, Shahsavar F (2018). Association of vitamin D level and vitamin D deficiency with risk of preeclampsia: a systematic review and updated meta-analysis. Taiwan J Obstet Gynecol.

[55] Ganguly A, Tamblyn JA, Finn-Sell S, Chan S-Y, Westwood M, Gupta J, Kilby MD, Gross SR, Hewison M. Vitamin D, the placenta and early pregnancy: effects on trophoblast function. J Endocrinol. 2018;236(2)R93-R103. 10.1530/JOE-17-0491 29109081

[56] Galinsky R, Polglase GR, Hooper SB, Black MJ, Moss TJM (2013). The consequences of chorioamnionitis: preterm birth and effects on development. J Pregnancy.

[57] Bastek JA, Weber AL, McShea MA, Ryan ME, Elovitz MA. Prenatal inflammation is associated with adverse neonatal outcomes. Am J Obstet Gynecol. 2014; 210(5):450.e451–450.410. 10.1016/j.ajog.2013.12.024 24361788

[58] Villamor-Martinez E, Álvarez-Fuente M, Ghazi AMT, Degraeuwe P, Zimmermann LJI, Kramer BW, Villamor E (2019). Association of Chorioamnionitis with Bronchopulmonary Dysplasia among Preterm Infants: a systematic review, meta-analysis, and Metaregression. JAMA Netw Open.

[59] Torchin H, Lorthe E, Goffinet F, Kayem G, Subtil D, Truffert P, et al. Histologic Chorioamnionitis and Bronchopulmonary dysplasia in preterm infants: the epidemiologic study on low gestational ages 2 cohort. J Pediatr. 2017;187. 10.1016/j.jpeds.2017.05.019 28583707

[60] Vrachnis N, Vitoratos N, Iliodromiti Z, Sifakis S, Deligeoroglou E, Creatsas G (2010). Intrauterine inflammation and preterm delivery. Ann N Y Acad Sci.

[61] Wang H, Xiao Y, Zhang L, Gao Q (2018). Maternal early pregnancy vitamin D status in relation to low birth weight and small-for-gestational-age offspring. J Steroid Biochem Mol Biol.

[62] Mousavi SE, Amini H, Heydarpour P, Amini Chermahini F, Godderis L (2019). Air pollution, environmental chemicals, and smoking may trigger vitamin D deficiency: evidence and potential mechanisms. Environ Int.

[63] Tamblyn JA, Hewison M, Wagner CL, Bulmer JN, Kilby MD (2015). Immunological role of vitamin D at the maternal-fetal interface. J Endocrinol.

[64] Roth DE, Morris SK, Zlotkin S, Gernand AD, Ahmed T, Shanta SS (2018). Vitamin D supplementation in pregnancy and lactation and infant growth. N Engl J Med.

[65] Adami S, Viapiana O Fau - Gatti D, Gatti D Fau - Idolazzi L, Idolazzi L Fau - Rossini M, Rossini M. Relationship between serum parathyroid hormone, vitamin D sufficiency, age, and calcium intake. (8756–3282 (Print)). 10.1016/j.bone.2007.10.003 18024243

[66] Smulian JC, Shen-Schwarz S, Vintzileos AM, Lake MF, Ananth CV (1999). Clinical chorioamnionitis and histologic placental inflammation. Obstet Gynecol.

[67] Steel JH, O'Donoghue K, Kennea NL, Sullivan MHF, Edwards AD (2005). Maternal origin of inflammatory leukocytes in preterm fetal membranes, shown by fluorescence in situ hybridisation. Placenta..

[68] Olmos-Ortiz A, Avila E, Durand-Carbajal M, Díaz L (2015). Regulation of calcitriol biosynthesis and activity: focus on gestational vitamin D deficiency and adverse pregnancy outcomes. Nutrients..

